# Virtual Reality and Web-Based Growth Mindset Interventions for Adolescent Depression: Protocol for a Three-Arm Randomized Trial

**DOI:** 10.2196/13368

**Published:** 2019-07-09

**Authors:** Jessica Lee Schleider, Michael C Mullarkey, John R Weisz

**Affiliations:** 1 Department of Psychology Stony Brook University Stony Brook, NY United States; 2 Department of Psychology University of Texas at Austin Austin, TX United States; 3 Department of Psychology Harvard University Cambridge, MA United States

**Keywords:** mental health, depression, virtual reality, adolescence, ehealth

## Abstract

**Background:**

Depression is the leading cause of disability in youth, with a global economic burden of US >$210 billion annually. However, up to 70% of youth with depression do not receive services. Even among those who do access treatment, 30% to 65% fail to respond and many dropout prematurely, demonstrating a need for more potent, accessible interventions. In a previous trial, a single-session Web-based growth mindset (GM) intervention significantly reduced depressive symptoms in high-symptom adolescents; however, this intervention did not benefit adolescents uniformly. For instance, the intervention reduced depression in adolescents who reported post intervention increases in perceived control, but it did not lead to significant depression reductions in adolescents who reported no significant post intervention increases in perceived control.

**Objective:**

The goal of this project is to test the acceptability and efficacy of a novel, single-session, virtual reality (VR) depression intervention—the *VR Personality Project*—teaching GM, the belief that personal attributes are malleable rather than fixed. The VR Personality Project was designed to systematically target and increase adolescents’ perceived control by offering a more immersive, engaging, user-directed intervention experience than the Web-based intervention can provide. By targeting an identified predictor of intervention response, the VR Personality Project may lead to larger reductions in depressive symptoms than existing Web-based mindset interventions.

**Methods:**

Adolescents with elevated depressive symptoms or a recent history of depression (N=159; ages 12 to 16 years) will be randomized to one of 3 intervention conditions: the VR Personality Project; the Web-based GM intervention tested previously; or an active, Web-based control. Adolescents and their parents will report on the adolescents’ depression symptoms, perceived control, and related domains of functioning at preintervention, postintervention, and at 3- and 9-month follow-up assessments.

**Results:**

We predict that the VR and Web-based mindset interventions will both lead to larger reductions in adolescent symptoms than the control intervention. Additionally, we predict that the VR-based single session intervention will lead to larger reductions in depression than the online mindset intervention and that these symptom reductions will be mediated by increases in adolescents’ perceived control from pre- to postintervention.

**Conclusions:**

The results may suggest an efficient strategy for reducing adolescent depressive symptoms: One that is mechanism-targeted, relatively affordable (less than US $200 for a commercially available VR headset, a fraction of the cost of long-term psychotherapy) and potentially engaging to adolescents experiencing mood-related distress.

**Trial Registration:**

ClinicalTrials.gov NCT0385881; https://clinicaltrials.gov/ct2/show/NCT03858881 (Archived by WebCite at http://www.webcitation.org/78C3roDgA).

**International Registered Report Identifier (IRRID):**

DERR1-10.2196/13368

## Introduction

### Background

Psychiatric disorders are the leading cause of disability worldwide, and 40.5% of this burden is attributable to depressive symptoms and disorder. Levels of depressive symptoms increase markedly in adolescence with nearly 20% of the youth experiencing a depressive disorder between ages 12 and 18 years [1]. Adolescent-onset depression accounts for 66% of lifetime depression cases and predicts interpersonal problems, substance abuse, and a 20-fold increased risk of attempting suicide. Despite this early onset and protracted course, up to 70% of adolescents with depression symptoms and disorders do not receive services [2-4]. Even among those who do access psychosocial or medication-based treatment, 30% to 65% fail to respond [5] and many drop out of clinic-based services prematurely—after 3.9 sessions on average [6]. These findings highlight the urgent need for more potent and accessible interventions for adolescent depression.

Emerging work suggests that single-session interventions (SSIs) can increase accessibility of potent interventions for youth depressive symptoms and disorders [7]. SSIs include core elements of comprehensive, evidence-based treatments, but their brevity makes them easier to disseminate to diverse settings. Indeed, SSIs can successfully treat youth psychiatric problems: In a meta-analysis of 50 randomized trials [4], SSIs for youth psychological problems demonstrated significant beneficial effects (mean *g*=.32) across various levels of youth problem severity, suggesting the potential of SSIs for youths with diagnosable and subclinical psychopathology. Furthermore, significant effects emerged even for self-administered (eg, Web-based) interventions (mean *g*=.32). Notably, SSIs’ overall effects are slightly smaller than those observed for multisession psychotherapy [8]. However, their high potential to render services more scalable and accessible—especially for youths who might otherwise go without services entirely—could magnify their benefits for youth psychological health on a large scale.

One SSI in particular has shown promise in reducing adolescent depressive symptoms: the growth mindset (GM) SSI, which encourages youths to view traits and attributes as malleable (a GM) as opposed to unchangeable (a *fixed mindset*). Youths holding fixed mindsets of personal traits tend to report higher levels of psychopathology [9] and increased internalizing problems over time [10,11]. Recent research suggests that encouraging GMs via brief, targeted interventions can help shift this trajectory: 30- to 90-min self-administered GM SSIs have prevented adolescent depression symptoms in nonclinical samples (OR=.55, with the GM group showing lower odds of reporting clinically elevated levels of depression 9 months later [12]). In a randomized controlled trial (RCT) targeting high-symptom adolescents, a GM SSI led to postintervention increases in adolescents’ perceived control over behavior (*d*=.34, *P*<.001) and emotions (*d*=.19, *P*=.03) relative to a comparison (supportive therapy [ST]) SSI [13]. The GM SSI also predicted steeper 9-month declines in youth depression symptoms per parent (*B*=–0.99, *P*=.047) and adolescent reports (*B*=–1.37, *P*=.03) [14].

Although multiple versions of GM SSIs have been developed to fit varying settings and populations, they are generally self-administered by youths; teach the *brain science* behind why it is possible for a trait (eg, personality, loneliness, and anxiety) to change; include testimonial quotes from peers reinforcing the possibility of personal change; and involve the completion of at least one *self-persuasion* exercise wherein youths write about how change is possible to help a peer who is struggling [15]. Thus, the GM SSI may help remove barriers to adolescents asking for help and for expanding effort rather than withdrawing in the face of setbacks and failure.

Despite its promising effects, it is notable that the GM SSI does not reduce depression in all adolescents. For instance, in the most relevant RCT, the GM SSI reduced depressive symptoms in adolescents who reported postintervention increases in perceived control over their personal behaviors, but it did not lead to significant depression symptom reductions in adolescents who reported small or no increases in perceived control [16]. Thus, the potency of GM SSIs for adolescent depression has yet to be optimized. Such potency may be advanced by developing new iterations of GM interventions (GMIs), which are designed to more systematically target predictors and mechanisms of clinical outcomes, such as low levels of perceived control: a predictor and risk factor for depression [17-21] that GM SSIs have successfully mitigated, both in the short-term [13] and over time [14]. Such efforts may increase the promise of GMIs to produce larger, longer lasting symptom reductions for a greater proportion of youth.

### Objectives

Accordingly, the goal of this 3-arm RCT is to evaluate the acceptability and efficacy of a novel, single-session virtual reality (VR)-based GM SSI—the *VR Personality Project* —for depressive symptoms in adolescents compared with both a Web-based GM-SSI and an active, Web-based control program. Immersive VR creates interactive, computer-generated worlds, which substitute real-world sensory perceptions with digitally generated ones, producing the sensation of actually being in new life-sized environments. The last 2 decades have seen a significant increase in the use of VR technology in mental health interventions, with research suggesting benefits of VR-mediated interventions for various anxiety disorders, specific phobias, posttraumatic stress disorder, substance use, and eating disorders [22-27], largely through graded exposure to feared stimuli and situations. VR has also been extended to the adjunctive treatment of psychotic symptoms, delivering cognitive rehabilitation, and social skills training interventions in ecologically valid virtual environments [28,29]. For this study, the VR Personality Project was designed in collaboration with Limbix Inc to systematically target and increase adolescents’ sense of perceived control by offering a more immersive, active, and user-directed intervention experience than Web-based GM SSIs have provided. Within the VR program, participants can exert control over their intervention experience by actively engaging with *characters* in the VR world, autonomously navigating through various environments and speaking directly to (eg, offering verbal advice) same-aged *peers*. By strengthening adolescents’ interactions with the program content, lessons, and characters and providing a more ecologically valid environment (relative to that offered by a computer-based program) for youths to rehearse and apply newly acquired skills, the *VR Personality Project* may engage an identified predictor of response to GM SSIs in turn producing larger reductions in depression than Web-based versions. Thus, the *VR Personality Project* may represent a mechanism-targeted, efficient strategy for reducing adolescent depression: one that is both relatively affordable (less than US $200 for any commercially available VR headset; a fraction of the cost of long-term psychotherapy) and potentially engaging to adolescents experiencing mood-related distress.

Notably, a recent systematic review of studies evaluating VR applications for mental health identified only 2 studies that have tested immersive VR mental health treatment approaches; both were uncontrolled feasibility trials targeting adults [19]. Thus, to our knowledge, this study will be the first randomized trial evaluating a brief VR intervention for adolescent depressive symptoms.

This research has 4 specific aims. Our first aim is to replicate past research suggesting that GM SSIs can significantly reduce depressive symptoms in at-risk adolescents. We hypothesize that adolescents aged 12 to 16 years who participate in a GMI (Web-based *or* VR–based) will show larger reductions in depression symptoms from baseline through the 9-month follow-up assessment compared with adolescents who receive an active, Web-based control program.

Our second aim is to evaluate new, single-session, VR GMI including a comparative efficacy study. Our second aim is to also evaluate whether the new VR–based GM SSI (the VR Personality Project) can reduce depressive symptoms in adolescents, both relative to an active control program and to the previously tested Web-based GM SSI [13,14]. We hypothesize that adolescents who participate in the VR–based GM SSI will show larger reductions in depressive symptoms from baseline through the 9-month follow-up assessment compared with adolescents who receive the Web-based GMI *and* compared with adolescents who receive the active Web-based control program.

Our third aim is to test whether shifts in perceived control mediate intervention effects on adolescent depressive symptoms. The VR Personality Project was designed to target and increase adolescents’ perceived control by offering a more immersive, active, and user-directed intervention experience than the Web-based GM SSI can provide. Thus, the third goal of this study is to examine whether the VR Personality Project does, in fact, reduce adolescent depressive symptoms by eliciting proximal increases in perceived control. We hypothesize that the VR Personality Project will lead to larger increases in immediate postintervention perceived control than the Web-based intervention from pre- to postintervention and that these increases will mediate subsequent reductions in adolescent depression across the follow up period.

Our fourth aim is to gauge acceptability of the VR intervention. Adolescents’ perceptions of any intervention can impact completion rates, program engagement, and ultimately intervention effectiveness. Thus, an additional aim of this research is to examine whether adolescents view the VR Personality Project as more engaging, helpful, and interesting than the Web-based GMI or the Web-based control intervention.

## Methods

### Summary of Overall Study Design

This study will be a 3-arm RCT, including 2 active intervention conditions and 1 active control condition. Study procedures were preregistered in ClinicalTrials.gov before enrollment of the first participant (NCT03858881; recruitment start date: March 2019). The Stony Brook University Institutional Review Board (IRB) has approved all study procedures described below. Participants will be randomly assigned to one of 3 conditions in equal numbers. We opted for equal allocation across groups (a relatively conservative approach) rather than weighted allocation to the active intervention groups, owing to the novelty of the VR intervention being evaluated and the resulting need for a rigorous, controlled test of its efficacy. After qualifying for participation based on a phone screen, adolescents (and 1 caregiver per adolescent) will visit the Department of Psychology at Stony Brook University for a 2-hour laboratory visit. Adolescents and parents will complete baseline questionnaires (see below for details). Adolescents will then be randomized to receive one of 3 interventions using a computer-based random number generator: the VR GM intervention (VR GMI), the online GM intervention (online GMI), or an online active control program designed to replicate ST and tested previously [13,14]. Immediately after intervention completion, adolescents will complete a postintervention questionnaire battery. Adolescents and parents will then be asked to complete online follow-up questionnaire batteries 3- and 9-month postintervention.

### Subjects, Projected Screen Failure Rate, and Power Analysis

We intend to recruit 159 adolescents aged 12 to 16 years (inclusive). G*Power 3.1 ( University of Duesseldorf) was used to calculate the sample size needed to achieve sufficient power, (1- β) to detect mean group differences (based on an omnibus *F* test) of small (.2), medium (.5), and large intervention effects (.8) on depression symptoms, measured continuously, with alpha=.05 and power at 0.80 for a 3-arm randomized trial. Sample sizes calculated were 969, 159, and 66 for effects of .2, .5, and .8, respectively, for an omnibus one-way analysis of variance (ANOVA). Power to detect a small effect size is ideal, but logistical constraints necessitate a more conservative sample size. The sample size of 159 (53 per SSI condition) reflects power to detect a medium (*d*=.5) between-group effect size.

### Detailed Study Procedures

#### Recruitment and Screening

Youth participants will be recruited from community groups, after-school and extracurricular programs, parent organizations, private psychiatric and pediatric primary care clinics, and religious organizations in the Stony Brook area. Eligibility for participation will be ascertained through a parent phone screen conducted by a trained member of the research team. Parents will be informed at the end of the phone screen whether or not their adolescent qualifies for study participation. Youths must be living with at least 1 parent or legal guardian and both must speak English well enough to complete study interventions. Additional inclusion criteria will include the following: (1) the youth is aged 12 to 16 years (inclusive) with 1 parent willing to participate (2) the youth reports elevated depressive symptoms (>80 percentile for age and sex, reflecting subclinical or higher symptom elevations) based on the parent-report version of the Children’s Depression Inventory-2 (CDI-2). Exclusion criteria will include intellectual disability (based on parent report) and hospitalization of the adolescent within the past 2 months for suicide attempt or self-harm, as the interventions being evaluated in this study are not designed for youth with acute medical or psychiatric need. Concurrent treatment will not preclude eligibility. Youths prone to seizures will also be eligible to participate; risks of participating will be discussed with prospective participants and families before study participation. This study will focus on youth aged 12 to 16 years because depression increases markedly in adolescence, and youth in this age range have responded well to GMI [13,14].

#### Laboratory-Based Study Session

On the basis of the parent phone screen, parents of eligible youths will be invited to schedule a laboratory-based study session, which they and their adolescent will attend together. This session will last approximately 2 hours and will be led by 2 research assistants at the postbaccalaureate, master’s, or advanced undergraduate level. Before guiding participants through the study, each research assistant will have received individual training from the principal investigator in each step of the study protocol, including 2 start-to-finish *practice runs* with mock participants.

At the start of this laboratory-based study session, the youth and parent will have the opportunity to provide consent or parental permission and youth assent; all study procedures will be explained to the family at this time, and the youth and parent will be reminded that they can choose to leave the study at any time. After providing parental permission and youth assent, study procedures will begin. The youth participant will be escorted to a separate room by a member of the study team to complete study procedures; the parent will remain in the room in which consent and assent was obtained to complete his or her portion of the study procedures (ie, a questionnaire battery). One member of the study team will remain with the youth for the duration of the session; a second member of the study team will provide instructions to the parent and remain available to answer any additional questions the parent has during the laboratory visit.

After consenting, youths will be asked to complete a battery of questionnaires (detailed below) via Qualtrics, a secure collection platform. Subsequently, a random number generator (embedded within the last slide of the Qualtrics survey including youths’ baseline questionnaires) will be used to assign youths to one of 3 intervention conditions: VR GMI, Web-based GMI, or Web-based ST. No personally identifying data or information will be collected during intervention administration.

Notably, for youths randomly assigned to the VR GMI, both the youth and experimenter will be aware of the condition assignment (only one of the 3 programs involves VR technology). However, for youths randomly assigned to either of the 2 Web-based interventions neither the youth nor the experimenter will be aware of which Web-based intervention they received; randomization to these 2 conditions will occur without experimenter involvement as part of the youth’s Qualtrics survey.

### Interventions

#### Web-Based Growth Mindset Intervention

The Web-based GMI [13,14], called Project Personality, is delivered via Qualtrics and takes approximately 30 min to complete. All intervention activities are self-administered by the youth and delivered in a Web-based format, including illustrations and audio-recordings of text. Intervention content is designed to maximize relevance for youths experiencing symptoms of depression, including excessive sadness and hopelessness. The intervention includes 5 components: (1) an introduction to the brain, including a lesson on the concept of neuroplasticity, describing how and why our behaviors are controlled by thoughts and feelings in their brains, which have potential for change; (2) written testimonials from older youths who describe their beliefs that people’s personal traits (eg, sadness and anxiety) are malleable, given the brain’s plasticity; (3) additional vignettes written by older youths, describing times when they used *GMs* to persevere through social and emotional setbacks; (4) a summary of scientific studies suggesting that personality can, and often does, change in positive ways over time; and (5) exercises in which the participants write notes to peers, drawing on scientific information to describe the malleability of people’s personal traits.

#### Virtual Reality Growth Mindset Intervention

The VR intervention, called the VR Personality Project, will be administered through an adjustable VR headset that includes a stereoscopic display powered by a Samsung smartphone (Galaxy S6TM) mounted on a lightweight (345 g) wireless, off-the-shelf head-mounted display with a 101-degree field of view for users (Samsung Gear Virtual Reality (VR)™). A focus wheel on the VR goggles will be adjusted to find a comfortable focal length for each participant. Sound is delivered through the head-mounted display. A lightweight (295 g) wireless Bluetooth controller (MOGA PROTM POWER controller) that requires only one hand to operate will be used by each adolescent to interact with the VR environment. For infection control reasons and because the same hardware will be used for all participants, disposable coverings will be used on the head-mounted display. All equipment will be cleaned between participants using disposable wipes and dried for at least 20 min.

**Figure 1:**
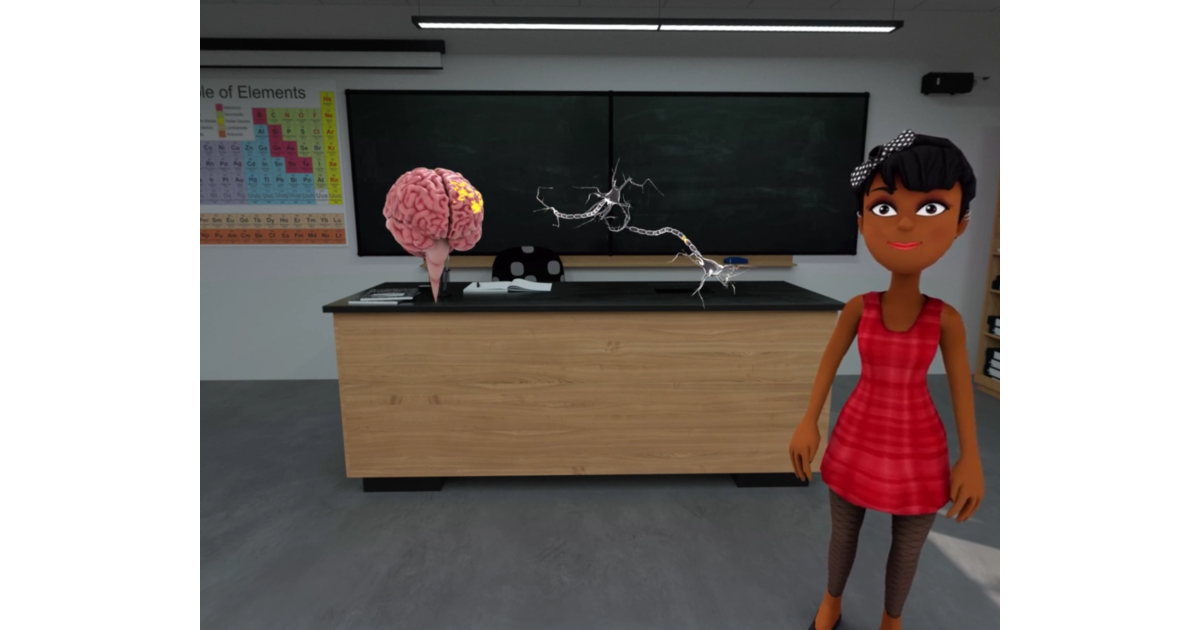
Screen Capture from VR Personality Project – lesson on neuroplasticity.

**Figure 2:**
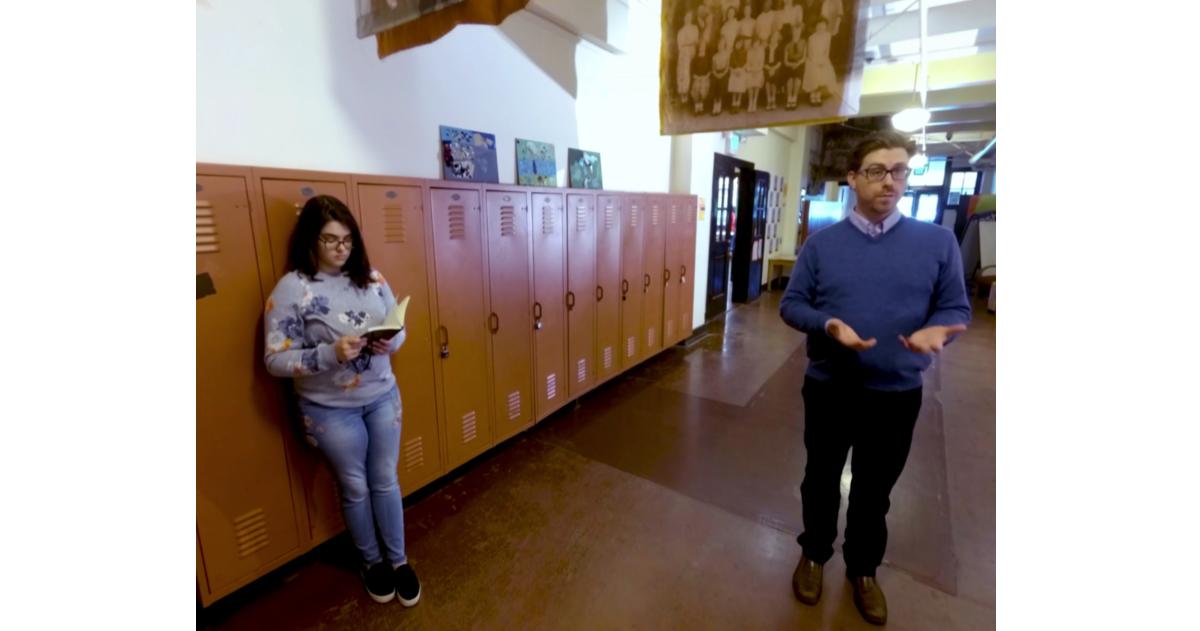
Screen Capture from VR Personality Project – a “scientist” describes research suggesting the malleability of personal traits.

**Figure 3:**
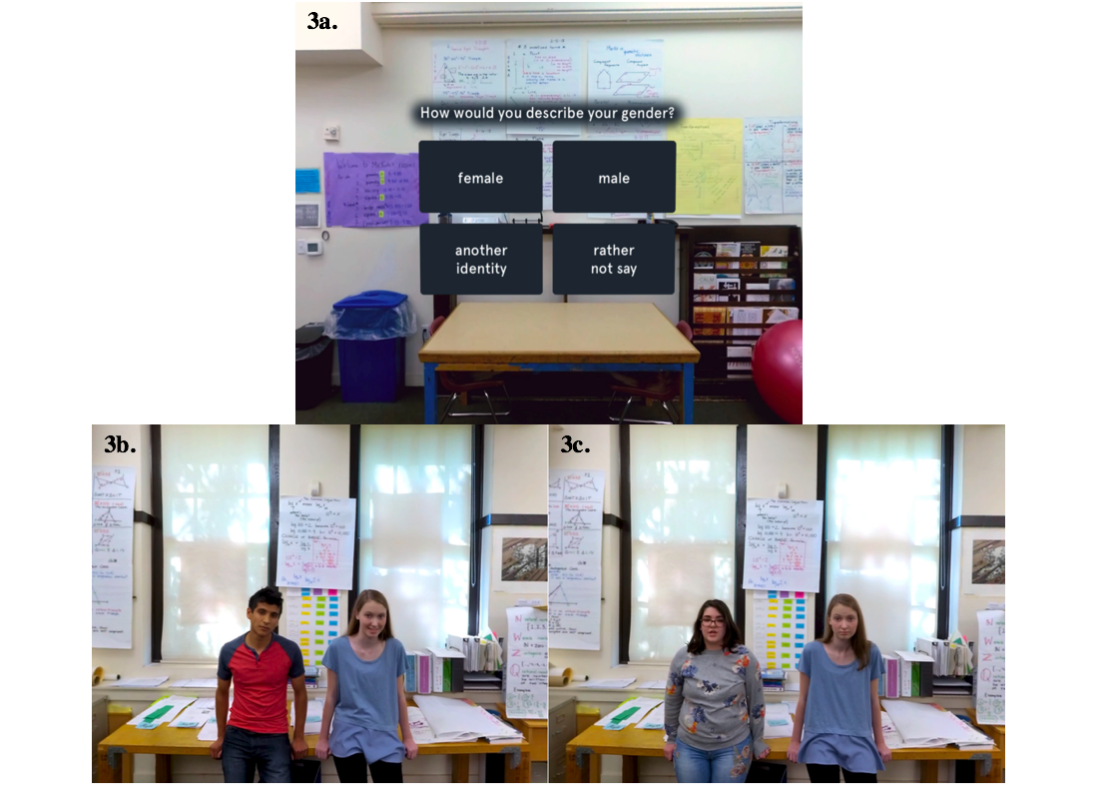
Screen Capture from VR Personality Project. 3a: Participants select gender identity. 3b: Peer guides for adolescents who are male-identifying. 3c: Peer guides for adolescents who are female-identifying. Adolescents who select “another identity” or “rather not say” encounter peer guides shown in panel 3b.

Similar to the Web-based analogue, the VR Personality Project takes approximately 30 min for youths to complete. It contains each of the components included in the Web-based GMI, including a lesson on neuroplasticity (Figure 1); testimonials from older peers; information about research suggesting the malleability of personal traits (Figure 2); and a self-persuasion exercise wherein the participant provides advice to a student in the VR environment who has just experienced a peer-related setback. Content is delivered by *characters* in the VR environment (adolescent and adult actors hired and filmed for the creation of this intervention), who are matched to an adolescent’s self-identified gender identity for a more personalized experience (Figure 3). These characters help guide the youth participant through each stage of the program, providing scientific information and personal stories. The primary difference between the VR and Web-based GMI involves the content delivery system and, by extension, the level of immersion each intervention offers. The VR program is designed to be immersive, fun, and interactive; the youth have an opportunity to choose to speak to various scientists and students within the VR environment and can navigate from one scene to the next; by contrast, in the online program, participants are automatically exposed to a series of text-based activities. Additionally, the VR Personality Projects provides participants the opportunity to provide advice (by speaking aloud to a student) in the VR environment, immediately after the student experiences a setback. Thus, the intervention offers a more self-directed, active experience for participants, as opposed to the passive experience of progressing through a largely text-based online program. Distinctions and similarities in user experience and content between the Web-based and VR GMIs are outlined in Table 1.

Youths who become dizzy or experience discomfort during the VR experience will be permitted to take breaks, or stop participating, at any time. Generally, glasses fit within the VR headset and may be worn during the intervention; however, the choice to wear or remove glasses (for participants who wear them) will be made on a person-to-person basis, based on personal preference and comfort. Youths will be reminded of this at the start of the laboratory session and again before starting the VR experience for those randomized to this condition.

**Table 1:** Similarities and differences between the virtual reality (VR) and computer-based personality project interventions.

Intervention design feature	Virtual reality personality project	Computer-based personality project
Delivery format	Immersive, 360-degree virtual reality environments	Computer-based program delivered via Qualtrics survey
Personalization of intervention content	Characters in program vary in age, gender identity by participant-selected age and gender identity	Static program content; no personalization by user characteristics
Degree of user choice	Participants select when to proceed to subsequent intervention sections; where to focus attention and which characters to speak to in a variety 360-degree environment; how many questions to ask program *characters*; and whether to engage in each intervention activity	Participants select when to proceed to subsequent intervention sections and whether listen to audio-recordings of written intervention text
Core program content	Lesson on neuroplasticity and the brain; psychoeducation regarding links between thoughts, feelings, and actions; peer and scientist testimonials, “saying-is-believing” activities to solidify learning. Delivered by characters and computerized animations in various VR environments.	Lesson on neuroplasticity and the brain; psychoeducation regarding links between thoughts, feelings, and actions; peer and scientist testimonials, “saying-is-believing” activities to solidify learning. Delivered via written text and accompanying audio-recordings of text.
Structure of “saying-is-believing” activity	Participants witness a program character undergo an in-vivo stressor related to depressive symptoms. Participants (a) describe how they themselves would feel, were they to experience a similar stressor, by speaking out-loud to the VR program guide, and (b) offer advice to help this character cope more effectively with the setback, again by speaking out-loud to the program character. Participants select when to begin and stop speaking.	Participants read about a peer undergoing a stressor related to depressive symptoms. Participants (a) write about how they themselves would feel, were they to experience a similar stressor, and (b) offer written advice to help this peer cope more effectively with the setback (using newly-gleaned knowledge about personality and the brain).

#### Web-Based Supportive Therapy

The Web-based ST [13,14] intervention, called the Sharing Feelings Program, is delivered entirely via Qualtrics, is self-administered by youths, and takes approximately 30 min to complete. It is structurally similar to the Web-based GMI, but it is designed to mimic ST. The goals of the ST intervention are to encourage youths to identify and express feelings to close others; the intervention does not teach or emphasize specific skills or beliefs. In a previous trial, ST led to smaller improvements in adolescent stress recovery, perceived control, and internalizing problems compared with a GM program [13,14]. The ST SSI is designed to control for nonspecific aspects of intervention, including engagement in a computer program. It includes the same number of reading and writing activities as the Web-based mindset intervention; it also mirrors the Web-based mindset intervention’s structure, including vignettes written by older peers describing times when they benefited from sharing feelings with close others. Immediately following intervention completion, all youths (regardless of condition assignment) will be asked to complete the same battery of questionnaires immediately postintervention to index immediate shifts in proximal outcomes.

After both the youth and the parent have completed their respective portions of the study, the laboratory visit will conclude. All study participants will be offered referral information for psychotherapy and/or pharmacologic treatment at Stony Brook and the surrounding community. Participants will not be excluded from completing study procedures if they begin receiving treatment for psychological distress during the study.

Before the youth and parent leave the laboratory, study personnel will inspect the youth and parent questionnaire responses on items inquiring about suicidal ideation. If there is any indication of adolescent suicidal ideation during the laboratory session, risk assessments will be conducted by trained study personnel. In the event that a participant is in imminent danger to themselves or others, their accompanying parent will be informed. Study personnel will meet with the family to establish a safety plan. In the event of reported, active suicidal ideation or plan, the family will be accompanied to the Psychiatric Emergency Program at the nearby University Hospital.

### Participant Incentives

Upon completing the 2-hour laboratory-based session, participating families will receive one US $30 Amazon gift card, for a rate of US $15 per hour, the standard rate approved by the University IRB and consistent with minimum wage in New York State. When each participating adolescent and parent complete the 3-month follow-up questionnaire, the family will receive a US $10 Amazon gift card, again based on a US $15 per hour rate. Similarly, when the adolescent and parent both complete the 9-month follow-up questionnaire, the family will receive a final US $10 Amazon gift card. Thus, total compensation for participating in this study is US $50.

### Follow-Up Assessments

To enable evaluation of the interventions’ effects on depressive symptoms and secondary study outcomes over time, each adolescent and parent will be invited to complete Qualtrics-based questionnaire batteries, including the same questionnaires as those they completed at their initial laboratory session. Links to Qualtrics surveys will be sent to families at 3- and 9-month follow-up points. Surveys may be conducted via phone at the family’s request. Families who do not complete the follow-up questionnaires within 3 days of receipt will receive up to 3 reminder messages from the research team to encourage survey completion.

Notably, logistical constraints necessitated our limiting the number of follow-up assessments in this study to 2 per family (at 3- and 9-month postintervention), and analyses were planned accordingly (see below for a more thorough description). We elected a final follow-up assessment at the 9-month mark to maintain consistency with previous trials of GMI [13,14].

After the study is complete, an aggregate results summary will be emailed to families. Condition assignment will be revealed at this time, and all youths will receive access to both Web-based interventions. Youths who did not receive the VR program will be invited to complete the intervention at Stony Brook University.

### Questionnaires

Table 2 displays a timeline of all study procedures, including points at which each questionnaire will be administered to parents and/or youths. All questionnaires are detailed below.

#### Family and Treatment History Questionnaire

Parents will report demographic, family, and other background information (eg, age, sex, race, childhood adversity exposure, and mental health treatment history). Parents will also complete the 4-item Pubertal Development Scale [30] with regard to their adolescent, given the well-documented effects of puberty on depression onset.

#### Children’s Depression Inventory-2

Adolescent depressive symptom severity will be assessed using the CDI-2 [31] child form (youth-report) and parent forms (parent-report). The CDI-2 is a reliable, valid measure of youth depression severity, normed for youth age and sex and yielding raw and *T* scores. Changes in youth-report CDI-2 scores from baseline to each of the follow-up assessments (3-month and 9-month) will serve as the primary index of intervention effects. Changes in parent-report CDI-2 scores from baseline to each of the follow-up assessments (3-month and 9-month) will serve as a secondary index of intervention effects. The CDI-2 will not be administered to youths or to parents immediately postintervention, as we do not expect depressive symptom change to occur within the span of 1 study session. Changes in scores on other assessments will serve as secondary outcomes.

#### Screen for Child Anxiety and Related Disorders

Given high comorbidity between depression and anxiety [32], anxiety symptoms will be assessed at baseline and all follow-ups (except postintervention) using the Screen for Child Anxiety and Related Disorders-Child and -Parent versions (SCARED-C/SCARED-P): a 41-item self-report measure [33,34]. Youths and parents, respectively, rate (0 to 2) the degree to which statements describing anxiety symptoms are true about them or their adolescent. Higher summed SCARED-C and SCARED-P total scores indicate greater adolescent anxiety severity.

#### Primary Control Scale for Children

The primary control scale for children (PCSC) [35] is a 24-item scale measuring youths’ perceived ability to influence or alter objective events or conditions through personal effort. Youth rate agreement with statements about their ability to exert primary control (eg, “I can do well on tests if I study hard” and “I can get other kids to like me if I try”). The PCSC has shown acceptable internal consistency, 6-month test-retest reliability, and inverse relations to adolescent depression severity.

#### Secondary Control Scale for Children

The secondary control scale for children (SCSC) [19] is a 20-item scale measuring youths’ perceived ability to shape the personal impact of objective conditions on oneself by adjusting oneself to fit those conditions. Youth rate agreement with items reflecting various kinds of secondary control such as adjusting cognition (“When something bad happens, I can find a way to think about it that makes me feel better”). The SCSC has shown acceptable reliability and validity in a large youth sample.

#### Implicit Personality Theory Questionnaire

The Implicit Personality Theory Questionnaire [36] asks the youth to rate the extent of their agreement with 3 statements linked to the malleability of personality, using a 1 to 7 Likert scale (eg, “Your personality is something about you that you can't change very much”). Higher mean scores on these 3 items indicate a stronger fixed personality mindset, a lower score indicates a stronger growth personality mindset. Both youths and parents will report their mindsets of personality in this study.

#### University of California Los Angeles Loneliness Scale

The UCLA Loneliness Scale [37] is a widely used self-report scale of loneliness in adolescents. The 20-item version will be used in this study. Adolescents rate how often they experience loneliness in various contexts (eg, “How often do you feel part of a group of friends?” and “How often do you feel there is no one you can turn to?”). Higher scores indicate higher levels of loneliness.

#### Beck Hopelessness Scale—Short Version

The Beck Hopelessness Scale (BHS)-4 [38] is a shortened version of the 20-item BHS [39] designed for brief psychological screening purposes. The 4 items on this measure are “My future seems dark to me”; “Things just won’t work out the way I want them to”; “There is no use in really trying to get something I want because I probably won’t get it”; and “I feel that the future is hopeless and that things cannot improve.” On each item, participants rate their agreement from 0 to 3, resulting in a maximum of 12 points in total (higher scores indicate higher levels of hopelessness). The short version of the BHS has high internal consistency (alpha=.85) and correlates highly with measures of depressive symptoms, as well as the full-length BHS, in large studies of clinical and community samples [40].

**Table 2:** Schedule of enrollment, interventions, and assessments.

Schedule			Study period					
			Enrollment	Allocation (baseline)	Post-allocation			Close-out (9 months)
					Intervention administration	Immediate post-intervention	3 months	
**Enrollment**								
	Eligibility screen		X	—^a^	—	—	—	—
	Informed consent/assent		X	—	—	—	—	—
	Allocation		—	X	—	—	—	—
**Interventions^b^**								
	VR GMI^c^		—	—	X	—	—	—
	Web-based GMI^d^		—	X	—	—	—
	Web-based ST^e^		—	X	—	—	—
**Assessments**								
	**Youth self-report**						
		Children's Depression Inventory 2-Youth	—	X	—	—	X	X
		Screen for anxiety related disorders-Youth	—	X	—	—	X	X
		Primary control scale for children	—	X	—	X	X	X
		Secondary control scale for children	—	X	—	X	X	X
		Beck Hopelessness Scale-Short form	—	X	—	X	X	X
		UCLA^f^ Loneliness Scale-Version 3	—	X	—	X	X	X
		Implicit personality theories questionnaire	—	X	—	X	X	X
		Attitudes toward therapy scale	—	X	—	X	X	X
		Program feedback scale	—	—	—	X	—	—
	**Parent report**							
		Family demographics, youth treatment history	—	X	—	—	—	—
		Children's depression inventory 2-parent	X	X	—	—	X	X
		Screen for anxiety related disorders–parent	—	X	—	—	X	X
		Implicit personality theories questionnaire	—	X	—	—	X	X
		Attitudes toward therapy scale	—	X	—	—	X	X
		Brief symptom inventory-18	—	X	—	—	X	X

^a^Not applicable.

^b^Each ~30 minutes in length; randomized to receive 1 of 3.

^c^VR GMI: virtual-reality growth mindset intervention.

^d^GMI: growth mindset intervention.

^e^ST: supportive therapy.

^f^UCLA: University of California Los Angeles.

#### Brief Symptom Inventory 18

The Brief Symptom Inventory (BSI)-18 [41] is a valid, reliable screening tool for adult (here, parental) psychological distress. Adult respondents rate endorsement of 18 physical and emotional complaints on a 0 to 4 Likert scale. The BSI-18 includes 3 subscales for somatic, anxiety, and depressive symptoms. The total sum score yields an additional total distress score.

#### Attitudes Toward Therapy

Attitudes toward psychotherapy [42] will be assessed using a single item for youth and parents: “Lots of kids deal with difficult emotions at one time or another. On a scale from 1 (not at all helpful) to 10 (extremely helpful), how helpful do you think therapy or counseling would be for you (your child) in coping with these kinds of problems?”

#### Program Feedback Scale (Designed for This Study)

To assess the acceptability of each intervention program, youths will be asked to complete questions regarding their experience with the intervention to which they were assigned. Questions inquire about how much they enjoyed the program; whether they understood the program; whether they would recommend the program to a friend; and whether they found the program easy to use. The Program Feedback Scale was developed specifically for this study; several items from the Scale were drawn from previous research [13,14] but others are new to this study and have not been used previously. All items are included in Textbox 1.

**Program Feedback Scale items. Items rated on a 0 (really disagree) to 4 (really agree) scale unless otherwise specified.**

- I enjoyed the program
- I understood the program
- This program was easy to use
- I tried my hardest during the program
- I think the program would be helpful to other kids my age
- I would recommend this program to a friend going through a hard time
- I agree with the program’s message
- What did you like about the program? Please share as many true thoughts and feelings as you would like (*free response*)
- What would you change about the program? Please share as many true thoughts and feelings as you would like (*free response*)

### Timeline for Data Collection and Results Reporting

Data collection began in March 2019 and is projected to be complete by December 2021. Thus, we intend to report results by Spring of 2022. Upon completion of data collection and publication of results, deidentified participant-level data will be made publicly accessible.

## Results

### Aim 1: Attempt to Replicate Past Research

We will use mixed-effects linear models to test the hypothesis that interventions teaching GM (either VR or Web-based) predict reductions in adolescent depressive symptom severity (primary study outcome), per both adolescent and parent reports, relative to Web-based ST. Our study design, including 3 assessment points is structured to allow for detection of linear intervention effects. We will run additional mixed-effects linear models to assess whether interventions teaching GM predict larger improvements in secondary study outcomes (perceived primary and secondary control, hopelessness, loneliness, attitudes toward psychotherapy, and parent psychopathology) versus Web-based ST. Intervention condition will be a binary predictor variable in these models, with the VR GMI and Web-based GMI groups collapsed into a single *GM* intervention group. Potential covariates will include family income, age, sex, and time (baseline, postintervention where applicable, and 3-month and 9-month follow-up); each possible covariate will be included in analyses if it shows a significant association with a model outcome at baseline or any follow-up point (such associations are unlikely to occur, given randomization procedures, but remain possible). Models will include a random intercept and slope, an autoregressive error structure, and use full information maximum likelihood (FIML) estimation to address missing data. We will create 2 orthogonal planned contrasts for testing intervention effects. One planned contrast will examine whether the 2 active GM conditions differ from the ST control condition, whereas the other planned contrast will examine whether the VR-based intervention outperforms the Web-based intervention. A significant (*P*<.05) interaction between the first contrast and time would indicate that interventions teaching GM predicted significantly different 9-month change in an outcome relative to the control ST condition. We will also replicate these analyses for the most central symptoms of adolescent depression identified in previous analyses (*sadness*, *self-hatred*, and *loneliness*) and use Holm-Bonferroni corrections for multiple comparisons.

### Aim 2: Evaluate New, Single-Session, Virtual Reality Growth Mindset Intervention

As for Aim 1, we will use mixed-effects linear models to test the hypothesis that VR-GMI predicts greater reductions in adolescent and parent-reported depressive symptom severity (primary study outcomes), as well as perceived primary and secondary control, hopelessness, loneliness, attitudes toward psychotherapy, and parent psychopathology (secondary outcomes), relative to (1) the Web-based GM program, (2) the control program, and (3) either program, when combined into a single, *non-VR intervention* group. Models will be structured as described in Aim 1. A significant (*P*<.05) interaction between the second orthogonal contrast mentioned in Aim 1 and time would indicate that interventions teaching GM predicted significantly different 9-month change in an outcome relative to the control ST condition. We will also replicate these analyses for the most central symptoms of adolescent depression identified in previous analyses (*sadness*, *self-hatred*, and *loneliness* [43]) and use Holm-Bonferroni corrections for multiple comparisons.

### Aim 3: Test Mediation Through Perceived Control

To test whether the VR intervention and the Web-based GM intervention reduces youth-reported depressive symptoms through proximal increases in perceived control, we will conduct multiple mediation analyses which involve simultaneous indirect effects by multiple variables [44]. This approach allows for both an analysis of the total indirect effect (the aggregate indirect effect of all the candidate mediators under investigation) and analyses of specific indirect effects (ie, of each mediator under investigation). For mediation models assessing perceived control, we will use postintervention data to assess candidate mediators and 9-month follow-up data to assess depressive symptoms. In each model, the predictor variable will be intervention condition (in Model 1, VR versus Web-based GM intervention; in Model 2, VR versus Web-based ST; and in Model 3, Web-based GM versus Web-based ST); the simultaneous mediator variables will be postintervention perceived behavioral control and postintervention perceived emotional control. We will use bias-corrected bootstrapping to test the significance of specific and total indirect effects within the mediation model. Bootstrapping has the advantage of high statistical power without assuming multivariate normality in sampling distributions, enabling parsimonious analysis of one or several candidate mediators [44,45]. The Iavaan package version 0.5-16 in R version 2.15.1, which is capable of testing both multiple and single mediator models using FIML estimation, will be used for mediation tests [46]. To test for indirect effects of candidate mediators, parameter estimates of total and specific indirect effects are generated, along with their CI, using 1000 to 20,000 random bootstrapped samples. We will specify 5000 resamples in this study per Preacher and Hayes’ [32] recommendations. If the 95% bias-corrected CI for a total indirect parameter estimate does not contain 0, then that indirect effect can be considered statistically significant, demonstrating mediation [44,45]. Using this approach, this study will be sufficiently powered to detect significant indirect effects in each model (bias-corrected CIs for a 2-mediator model show .80 power for samples of 50 and over .90 for samples of 100 [46,47]).

### Aim 4: Gauge Acceptability of the Virtual Reality Intervention

A series of between-group ANOVAs will be used to evaluate differences by intervention condition assignment in adolescent-reported intervention acceptability. Specifically, we will examine group-level differences in adolescents’ mean ratings on each continuously rated item from the Program Feedback Scale. Specific contrasts comparing groups will be examined, should significant overall mean differences emerge on any item.

## Discussion

The objective of this 3-arm randomized trial is to evaluate whether a single-session, immersive VR intervention teaching *GM* —the belief that personal traits and attributes are malleable as opposed to fixed—can reduce depressive symptoms in high-risk adolescents compared with a Web-based GMI and an active Web-based control. Secondary aims are to evaluate the VR program’s effects on other types of adolescent symptoms and functioning, including anxiety, perceived control, and hopelessness; to evaluate a possible mechanism through which the VR might reduce depression symptoms (ie, by increasing adolescents’ sense of perceived control); and to assess the VR program’s acceptability relative to the Web-based interventions. Results will gauge the promise of the *VR Personality Project* as a brief, highly engaging, and mechanism-targeted intervention for reducing adolescent depressive symptoms. Given the increasing levels of adolescent depressive symptoms in recent years [2] and variable efficacy and accessibility of existing interventions [3,8], results of this trial may suggest a promising new approach, using immersive VR technology, to reducing depression in youth.

## Abbreviations

ANOVA: analysis of variance
BHS: Beck Hopelessness Scale
BSI: Brief Symptom Inventory
CDI-2: Children’s Depression Inventory-2
FIML: full information maximum likelihood
GM: growth mindset
GMI: growth mindset intervention
IRB: Institutional Review Board
PCSC: primary control scale for children
RCT: randomized controlled trial
SCARED-C: Screen for Child Anxiety and Related Disorders-Child
SCARED-P: Screen for Child Anxiety and Related Disorders-Parent
SCSC: Secondary Control Scale for Children
SSI: single-session intervention
ST: supportive therapy
VR GMI: virtual-reality growth mindset intervention
VR: virtual reality
